# The detection of sentinel lymph nodes in laparoscopic surgery can eliminate systemic lymphadenectomy for patients with early stage endometrial cancer

**DOI:** 10.1007/s10147-017-1196-9

**Published:** 2017-11-02

**Authors:** Tomohito Tanaka, Yoshito Terai, Satoe Fujiwara, Yoshimichi Tanaka, Hiroshi Sasaki, Satoshi Tsunetoh, Kazuhiro Yamamoto, Takashi Yamada, Masahide Ohmichi

**Affiliations:** 1 Department of Obstetrics and Gynecology, Osaka Medical College, 2-7, Daigaku-machi, Takatsuki, Osaka 569-8686 Japan; 2 Department of Radiology, Osaka Medical College, Takatsuki, Japan; 3 Department of Pathology, Osaka Medical College, Takatsuki, Japan

**Keywords:** Endometrial cancer, Sentinel lymph node, Laparoscopic surgery, Pelvic lymph node, Lymph node metastasis, Lymphadenectomy

## Abstract

**Background:**

The examination of a sentinel lymph node (SLN), where lymph node metastasis first occurs, may be advocated as an alternative staging technique. The aim of this study was to evaluate the feasibility and detection rates of an SLN biopsy in patients with endometrial cancer.

**Study design:**

Two hundred and eleven patients with endometrial cancer underwent an SLN biopsy at hysterectomy using three kinds of tracers including 99m-technetium-labeled tin colloid (^99m^Tc), indigo carmine and indocyanine green. Factors related to the side-specific detection rate, sensitivity and false negative rate were analyzed.

**Results:**

The detection rates of the SLN biopsy using ^99m^Tc, indigo carmine and indocyanine green were 77.9, 17.0 and 73.4%, respectively. The detection rate was lower in elderly patients (≥60 years) (67.9 vs 89.2%, *p* < 0.01), patients with >50% myometrial invasion (68.3 vs 85.2%, *p* < 0.01), patients with high-grade tumors (69.5 vs 84.9%, *p* < 0.01) and patients who underwent laparotomy (71.2 vs 84.9%, *p* < 0.01). There were no significant differences in body mass index. The sensitivity was not significantly different in any factor. However, the false negative rate was higher in patients with > 50% myometrial invasion (11.5 vs 1.2%, *p* < 0.01), high-grade tumors (13.3 vs 0.8%, *p* < 0.01) and who underwent laparotomy (12.2 vs 0.4%, *p* < 0.01).

**Conclusion:**

Patients who underwent laparoscopy with < 50% myometrial invasion and low-grade tumors not only have higher detection rates, but also have lower false negative rates. These patients may avoid systemic lymphadenectomy according to the status of the SLN biopsy.

## Introduction

Pelvic lymph node dissection (PLND) remains an important surgical procedure for treating endometrial cancer. This technique has resulted in a favorable prognosis [1–6], as well as correct staging [7–9], in patients with endometrial cancer. However, the rate of metastasis to pelvic lymph nodes is low among patients with low-risk cancer [10]. Furthermore, as surgical complications including nerve or vessel injury and lymph edema may occur [11–13], PLND may not be necessary for patients with low-risk endometrial cancer. Generally, the surgical method is determined according to the pre- or intraoperative status of the cancer. For patients with endometrial cancer, the extent of lymph node dissection is determined according to myometrial invasion and tumor grade on preoperative magnetic resonance imaging, preoperative biopsy and intraoperative frozen sections. However, the accuracy of these examinations is not sufficient for decision-making of PLND [14–17]. For these reasons, a sentinel lymph node (SLN) biopsy has gained attention. The SLN mapping technique is based on the principle that the first nodal group receiving lymphatic drainage from a primary tumor can be identified. The utility of SLN mapping has been well established in melanoma, breast cancer, and vulvar cancer [18–20].When an SLN can be intraoperatively diagnosed using frozen sections, systematic PLND may be avoided. An SLN algorithm is now included in The National Comprehensive Cancer Network (NCCN) guidelines for endometrial carcinoma with category 3 evidence [21, 22]. In the past decade, laparoscopic surgery has been performed increasingly frequently compared with open surgery for early stage endometrial cancer [23]. Laparoscopic surgery is associated with a quick recovery, early immobilization, minimal blood loss, less pain, and reduced need for analgesia and anticoagulants without a lengthier operation time [24]. However, few studies have examined the SLN detection rates among different surgical procedures (such as open surgery or laparoscopic surgery), and the outcomes of patients with uterine endometrial cancer, the most suitable tracer, the best injection site, and the indications for uterine endometrial cancer have not been standardized. In this study, we describe the diagnostic accuracy of an SLN biopsy in patients with endometrial cancer.

## Materials and methods

### Participants

Between September 2012 and June 2017, a total of 211 Japanese endometrial cancer patients underwent a sentinel node procedure at Osaka Medical College in Japan. All of the patients underwent laparoscopic or abdominal hysterectomy, bilateral salpingo-oophorectomy and an SLN biopsy with or without PLND and paraaortic lymph node dissection (PAND). Patients were eligible for SLN biopsy if they met the following criteria—(1) the preoperative diagnosis indicated that the patient did not have extrauterine disease (except for lymph node metastasis), and (2) the patient gave written informed consent for participation. When the patients did not have any serious complications, systematic PLND was performed after SLN biopsy. Laparoscopic procedures were indicated in cases involving low-risk disease (including < 50% myometrial invasion, with grade 1 or 2 endometrioid carcinoma). In contrast, laparotomic procedures with systematic PAND were indicated for patients with high-risk disease (including > 50% myometrial invasion or high-grade disease). When the intraoperative diagnosis revealed that the patient had extrauterine disease during the laparoscopic procedure, conversion to laparotomy was made and systematic PAND was performed. When the intraoperative diagnosis revealed that the patient had > 50% myometrial invasion, high-grade disease or SLN metastasis, systematic PAND was performed with laparoscopy or laparotomy. The present study was approved by the institutional review board and the participants provided their informed consent.

### SLN mapping procedure

All of the tracers were sub-mucosally injected in four quadrants of the cervix at 0, 3, 6, and 9 o’clock. The cervical injection was approximately 5 mm in all cases, as described previously [25–27]. On the day before the operation, 2.0 ml of fluid containing 110 MBq 99m-Technetium (^99m^Tc)-labeled tin colloids was injected into the patient’s cervix. Lymphoscintigraphy was performed within 6 h, and hot spots, indicating SLNs, were identified. On the day of the operation, 5 ml of indigo carmine (IDC) (2 mg/ml) and/or indocyanine green (ICG) (50 µg/ml) was injected into the cervix at the start of surgery. The same quantity of IDC and/or ICG was also injected into the uterine fundus upon reaching the intra-abdominal cavity. The SLN was detected at 40 min after injection of IDC or ICG. Radioactive lymph nodes were located using a gamma-probe (Navigator GPS; RMD). IDC-stained lymph nodes were detected by direct inspection. ICG fluorescence-positive lymph nodes were detected using a color fluorescence camera (Hyper Wye Medical System, Mizuho Co., for laparotomy; Camera Control Unit JC300, MC Medical Co., for laparoscopy). After the SLN biopsy, the area of pelvic lymph node was surveyed by direct observation, and with a color fluorescence camera or a gamma-probe to confirm that no radioactive tissue remained. A combination of ^99m^Tc and IDC was used in the early phase and a combination of the three tracers was used in the late phase.

### Pathology and SLN analyses

An intraoperative pathological examination was performed. The SLN was cut in half, parallel to the longest axis, to obtain a maximal section area. One half was used to create a frozen section. The specimen was cut with an interval of 2 mm, and a 5-µm section, which was stained with hematoxylin and eosin (H&E), was evaluated at the time that the frozen section was created. The other half and the non-SLN specimens were fixed in 10% formalin for a permanent section procedure; the specimen was cut parallel to the longest axis with an interval of 2 mm. After fixation, the 5-µm thick sections were stained with H&E and examined.

### Detection rate, sensitivity, and false negative (FN) rate

**Fig. 1:**
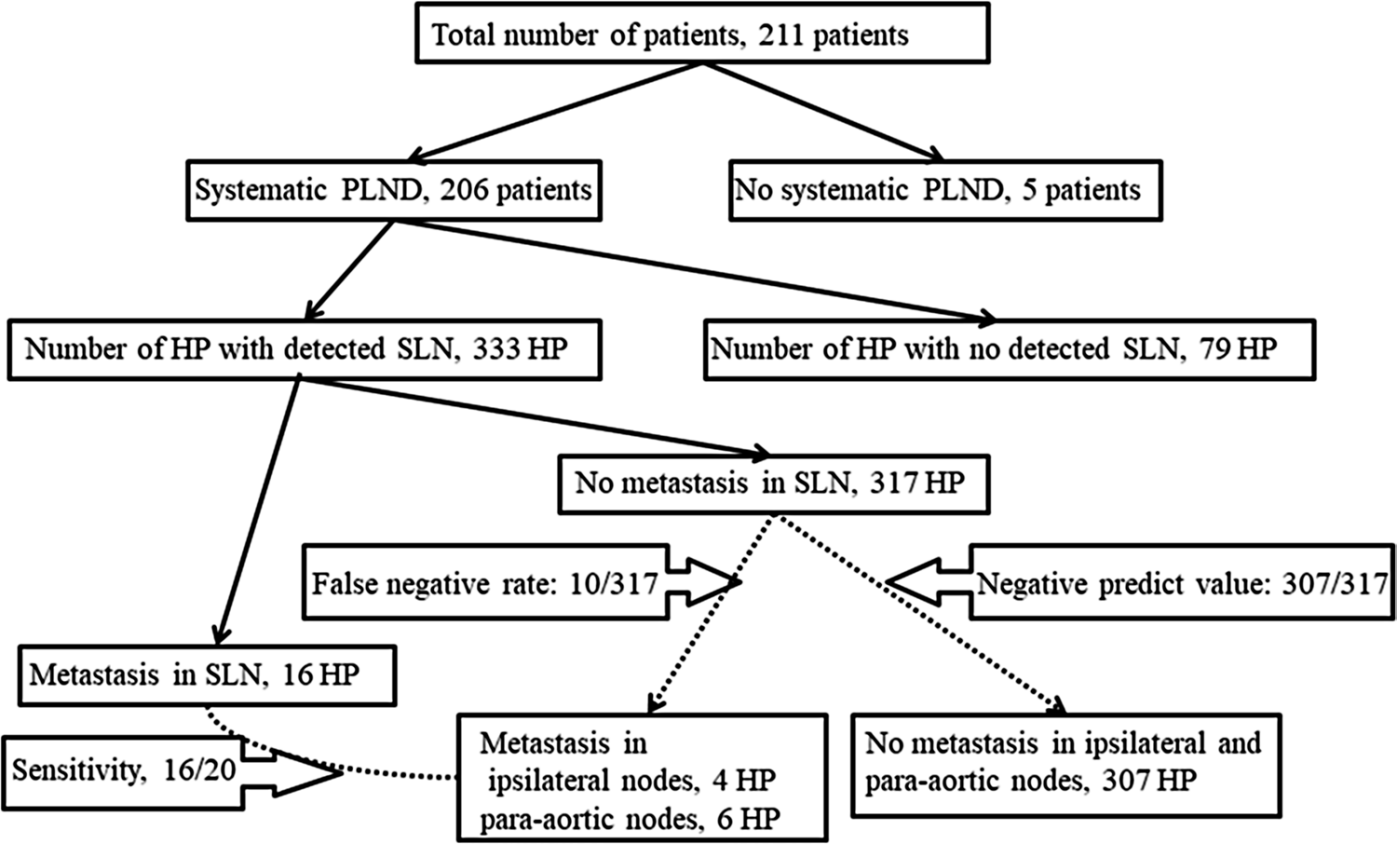
Calculation of the sensitivity, false negative (FN) rate, and negative predict value in the study participants. The calculations of each rate were side-specific. Among all 211 patients, 206 had systematic pelvic lymph node dissection (PLND). Among these patients, sentinel lymph nodes (SLNs) were detected in 333 hemi-pelvises (HP). Metastases was identified in 16 SLNs, with no metastasis identified in 317 HP. However, four metastases were identified in the ipsilateral nodes and six metastases were identified in para-aortic lymph nodes with no SLN metastasis (FN rate 10/317). No metastases were identified in the ipsilateral nodes or para-aortic nodes in the remaining 307 HP (negative predict value 307/317). Among the 20 HP with metastatic pelvic nodes, 16 SLNs had metastasis (sensitivity 16/20)

Figure 1 shows the calculations of the sensitivity, FN rate, and negative predictive value in the study participants. Each side of the pelvis was measured as a unit (the right and left pelvic regions were analyzed separately). The hemi-pelvises (HP) included the common iliac nodes, external iliac nodes, internal iliac nodes, and obturator nodes. The detection rate was defined as the ratio of the number of HP with at least one detected SLN to all HP. The sensitivity was defined as the ratio of the number of HP with at least one involved SLN to the number of HP with at least one involved node among the HP with at least one detected SLN. The FN rate was defined as ipsilateral pelvic lymph node metastasis or para-aortic lymph node metastasis without SLN metastasis; the FN rate was defined as the ratio of the number of HP with an FN case of SLN biopsy to the number of patients with at least one involved node, SLN or not, among HP with at least one detected SLN.

### Statistical analysis

Each rate including the detection rate, sensitivity and FN rate were calculated according to the HP. All of the statistical analyses were performed using the JMP software package (version. 11.1.1). Continuous variables are expressed as the mean ± standard deviation. The Mann–Whitney *U* test was used to compare continuous variables, and Fisher’s exact test was used to compare frequencies. A logistic regression model was used for the multivariate analysis. *P* values of < 0.05 were considered to indicate statistical significance.

## Results

**Fig. 2:**
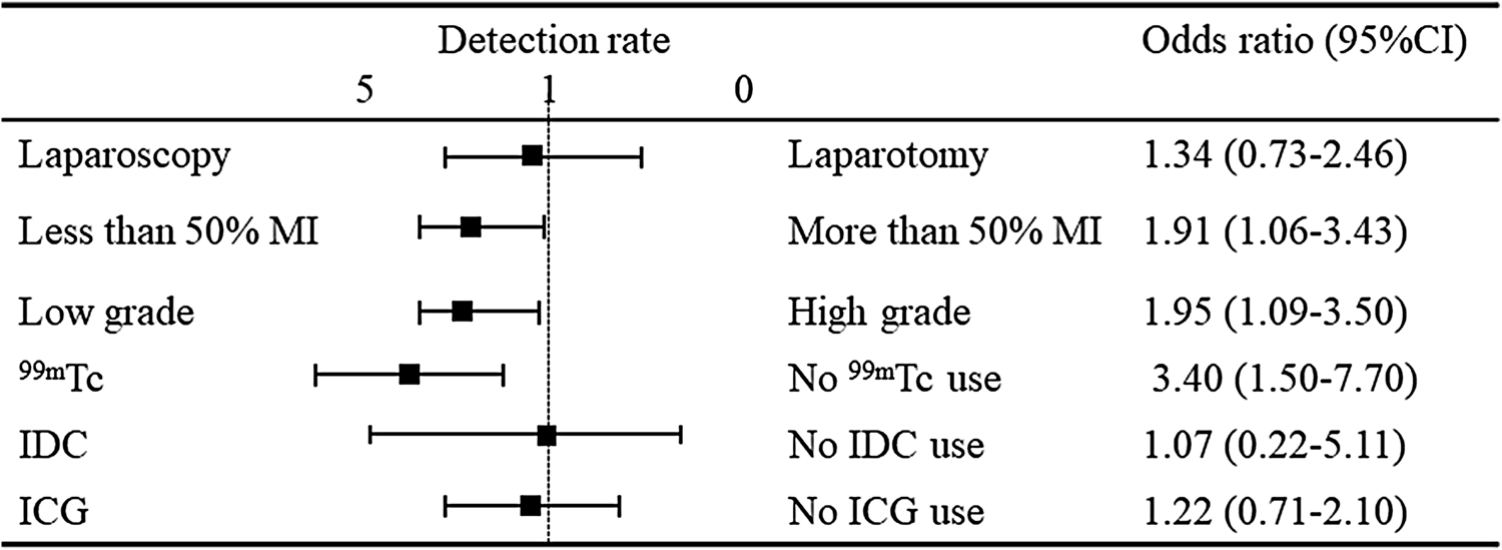
Multivariate analysis showed that < 50% myometrial invasion (MI), low-grade tumors and 99m-technetium-labeled tin colloid (^99m^Tc) use were independently associated with detection. However, laparoscopy, indigo carmine (IDC) and indocyanine green (ICG) use were not independently associated with detection

**Fig. 3:**
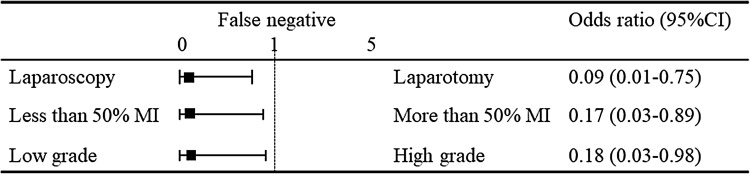
Multivariate analysis showed that laparoscopy, < 50% myometrial invasion (MI) and low-grade tumors were independently associated with the FN rate

Table 1 shows the characteristics of 211 patients with uterine endometrial cancer who underwent an SLN biopsy. The mean (± standard deviation, SD) age of the patients was 57.5 ± 11.1 years, and the mean body mass index (BMI) was 23.7 ± 4.7. Sixty-three (29.9%) patients were nulliparous. A total of 149 patients had FIGO stage IA disease, 26 had stage IB disease, 2 had stage II disease, 10 had stage IIIA disease, 2 had stage IIIB, 18 had stage IIIC and 4 stage IVB disease. Histologically, 159 patients had grade 1 or 2 endometrioid carcinoma, 24 had grade 3 cancer, 16 had serous carcinoma, 6 had clear cell carcinoma and 6 had carcinosarcoma. The SLN procedure was performed in 195 (92.4%) patients with ^99m^Tc tin colloid, 203 (96.2%) patients with IDC and 122 (57.8%) with ICG. Two hundred and six (97.6%) patients underwent systematic PLND after the SLN biopsy. Forty-nine (23.2%) patients underwent systematic PAND after the SLN biopsy. Fourteen (6.6%) patients underwent a para-aortic lymph node biopsy as SLN. These SLNs in the para-aortic node were located in the b2 area. The mean number of detected SLNs was 2.6 ± 1.7, while the mean numbers of SLNs detected with ^99m^Tc, IDG and ICG were 2.3 ± 1.7, 0.4 ± 0.8 and 2.2 ± 1.7, respectively. The total number of resected lymph nodes was 42.3 ± 21.3. One hundred and fifty-two patients underwent laparoscopic surgery and 59 underwent laparotomy. The mean age, BMI, and the rate of nulliparity did not differ to a statistically significant extent between the groups. The rate of patients with IA disease was higher in the laparoscopic group than in the laparotomic group (72.0 vs 42.4%, *p* < 0.01). The rate of IIIC disease was lower in the laparoscopic group (2.6 vs 23.7%, *p* < 0.01). The rate of low-grade tumors, including grade 1 or 2 endometrioid carcinoma was higher in the laparoscopic group (84.9 vs 50.8%, *p* < 0.01). In contrast, the rate of high-grade tumors (including grade 3 endometrioid carcinoma, serous carcinoma, clear cell carcinoma, and carcinosarcoma) was lower in the laparoscopic group. ICG was used more frequently in the laparoscopic group (47.1 vs 33.9%, *p* < 0.01). The rate of systematic PAND was significantly lower in the laparoscopic group (12.5 vs 50.8%, *p* < 0.01). The number of detected lymph nodes was significantly greater in the laparoscopic group (2.8 ± 1.8 vs 2.0 ± 1.2, *p* < 0.01). This was apparent with ICG (2.5 ± 1.7 vs 1.0 ± 1.0, *p* < 0.01). Figure 1 shows the calculation of the sensitivity, FN, and negative predictive value in the study participants. The calculations of each rate were side-specific. Among all 211 patients, 206 had systematic PLND. Among these patients, SLNs were detected in 333 HP. Metastasis was identified in 16 SLNs, with no metastasis identified in 317 HP. However, four metastases were identified in the ipsilateral lymph nodes and six metastases were identified in para-aortic lymph nodes in 317 HP with no SLN metastasis (FN rate 10/317). No metastases were identified in the ipsilateral nodes in the remaining 307 HP (negative predictive value 307/317). Among the 20 HP with metastatic lymph nodes, 16 SLNs had metastasis (sensitivity 16/20). Table 2 shows the detection rate, sensitivity and FN associated with related factors. Each rate was calculated according to the HP. The detection rate was calculated in all 211 patients with 422 HP. The sensitivity and FN were calculated in 206 patients with systematic PLND and 333 HP with detected SLN. The total detection rate, sensitivity and FN were 81.0, 80.0 and 3.2%, respectively. Elderly patients (≥ 60 years) had a lower detection rate than younger (<60 years) patients (67.9 vs 89.2%, *p* < 0.01). However, the sensitivity and FN were not significantly different (sensitivity 100 vs 66.7%, *p* = 0.07; FN 1.0 vs 4.1%, *p* = 0.1). The rates were not significantly different between obese (BMI ≥25) and non-obese (BMI < 25) patients (detection rate 82.3 vs 80.5%, *p* = 0.7; sensitivity 90.0 vs 70.0%, *p* = 0.3; FN 3.1 vs 3.2%, *p* = 0.9). Additionally, multiparous patients had a lower detection rate than nulliparous patients (78.4 vs 87.3%, *p* = 0.03). The sensitivity and FN were not significantly different between nulliparous patients and multiparous patients (sensitivity 75.5 vs 87.2%, *p* = 0.5; FN 3.2 vs 3.0%, *p* = 0.9). Patients with >50% myometrial invasion had lower detection rates (68.3 vs 85.2%, *p* < 0.01) and higher FN (11.5 vs 1.2%, *p* < 0.01) than those with <50% myometrial invasion. The sensitivity was not significantly different (81.8 vs 77.8%, *p* = 0.8). Patients with high-grade tumors, including grade 3 endometrioid carcinoma, serous carcinoma, clear cell carcinoma and carcinosarcoma had lower detection rates (69.5 vs 84.9%, *p* < 0.01) and higher FN (13.3 vs 0.8%, *p* < 0.01) than patients with low-grade tumors, including grade 1 or 2 endometrioid carcinoma. The sensitivity was not significantly different (76.9 vs 85.7%, *p* = 0.6). Among the three tracers, ^99m^Tc had a higher detection rate than IDC (77.9 vs 17.0%, *p* < 0.01). There was no significant difference between ^99m^Tc and ICG (77.9 vs 73.4%, *p* = 0.3). The sensitivity and FN rate were not significantly different between the tracers. The detection rates of the single, double and triple tracers were 25% (4/16), 84.2% (165/196) and 82.4% (173/210), respectively. Moreover, the laparoscopic procedure had a higher detection rate and lower FN rate than laparotomy (detection rate 84.9 vs 71.2%, *p* < 0.01; FN 0.4 vs 12.2%, *p* < 0.01). The sensitivity was not significantly different between laparoscopic procedures and laparotomy (85.7 vs 76.9%, *p* = 0.6). Figure 2 shows the results of the multivariate analysis of the factors associated with the detection rate. Myometrial invasion <50% (adjusted odds ratio [aOR] 1.91, 95% confidence interval [CI] 1.06–3.43), low-grade tumors (aOR 1.95, 95% CI 1.09–3.50), and ^99m^Tc use (aOR 3.40, 95% CI 7.70) were independently associated with detection. Laparoscopy (aOR 1.34, 95% CI 0.73–2.46), IDG (aOR 1.07, 95% CI 0.22–5.11), and ICG (aOR 1.22, 95% CI 0.71–2.10) use were not independently associated with detection. Figure 3 shows the results of the multivariate analysis of factors associated with the FN rate. Laparoscopy (aOR 0.09, 95% CI 0.01–0.75), <50% myometrial invasion (aOR 0.17, 95% CI 0.03–0.89) and low-grade tumors (aOR 0.18, 95% CI 0.03–0.98) were independently associated with the FN rate.

**Table 1:** Characteristics of patients with endometrial cancer who underwent sentinel lymph node biopsy

Total no. of patients	Total (*n* = 211)	Laparoscopy (*n* = 152)	Laparotomy (*n* = 59)	*p* value
Age^a^ (years)	57.5 ± 11.1	57.1 ± 11.2	58.5 ± 10.8	0.3
BMI	23.7 ± 4.7	23.7 ± 4.6	23.7 ± 4.9	0.9
Nulliparous (%)	63 (29.9)	103 (67.8)	45 (76.3)	0.08
FIGO stage (%)				
IA	149 (70.6)	124 (72.0)	25 (42.4)	< 0.01
IB	26 (12.3)	14 (9.2)	12 (20.3)	0.3
II	2 (0.9)	0	2 (3.4)	0.02
IIIA	10 (4.7)	6 (3.9)	4 (6.8)	0.4
IIIB	2 (0.9)	2 (1.3)	0	0.3
IIIC	18 (8.5)	4 (2.6)	14 (23.7)	< 0.01
IVB	4 (1.9)	2 (1.3)	2 (3.4)	0.3
Histological type (%)				
Endometrioid grade 1 or 2	159 (75.4)	129 (84.9)	30 (50.8)	< 0.01
Endometrioid grade 3	24 (11.4)	11 (7.2)	13 (22.0)	< 0.01
Serous carcinoma	16 (7.6)	6 (3.9)	10 (16.9)	< 0.01
Clear cell carcinoma	6 (2.8)	4 (2.6)	2 (3.4)	0.7
Carcinosarcoma	6 (2.8)	2 (1.3)	4 (6.8)	0.04
Tracers (%)				
^99m^Tc	195 (92.4)	141 (92.8)	54 (91.5)	0.8
IDG	203 (96.2)	145 (95.4)	58 (98.3)	0.3
ICG	122 (57.8)	102 (47.1)	20 (33.9)	< 0.01
Surgical method (%)				
Systematic PLND	206 (97.6)	147 (96.7)	59 (100)	0.1
Systematic PAND	49 (23.2)	19 (12.5)	30 (50.8)	< 0.01
PAN biopsy as SLN	14 (6.6)	12 (7.9)	2 (3.4)	0.2
No. of detected sentinel lymph nodes^a^	2.6 ± 1.7	2.8 ± 1.8	2.0 ± 1.2	< 0.01
^99m^Tc	2.3 ± 1.7	2.5 ± 1.7	1.8 ± 1.1	0.01
IDG	0.4 ± 0.8	0.5 ± 0.9	0.3 ± 0.1	0.1
ICG	2.2 ± 1.7	2.5 ± 1.7	1.0 ± 1.0	< 0.01
Resected lymph nodes^a^	42.3 ± 21.3	38.1 ± 16.1	54.1 ± 27.0	< 0.01

^*99m*^ *Tc* 99m-technetium-labeled tin colloid, *IDC* indigo carmine, *ICG* indocyanine green, *PLND* pelvic lymph node dissection, *PAND* para-aortic lymph node dissection

^a^According to ANOVA (mean ± SD)

**Table 2:** Association between detection rate, sensitivity and FN rate of sentinel lymph node biopsy and related factors

Factors	Detection rate (%)	*p* value	Sensitivity (%)	*p* value	False negative (%)	*p* value
Total	342/422 (81.0)		16/20 (80.0)		10/317 (3.2)	
Age (years)						
< 60	232/260 (89.2)		8/12 (66.7)		9/218 (4.1)	
≥ 60	110/162 (67.9)	< 0.01	8/8 (100)	0.07	1/99 (1.0)	0.1
BMI						
< 25	235/292 (80.5)		7/10 (70.0)		7/220 (3.2)	
≥ 25	107/130 (82.3)	0.7	9/10 (90.0)	0.3	3/97 (3.1)	0.9
Parity						
0	110/126 (87.3)		7/8 (87.2)		3/99 (3.0)	
≥ 1	232/296 (78.4)	0.03	9/12 (75.5)	0.5	7/218 (3.2)	0.9
Myometrial invasion						
< 50%	271/318 (85.2)		7/9 (77.8)		3/256 (1.2)	
≥ 50%	71/104 (68.3)	< 0.01	9/11 (81.8)	0.8	7/61 (11.5)	< 0.01
Tumor grade						
Low	269/317 (84.9)		6/7 (85.7)		2/257 (0.8)	
High	73/105 (69.5)	< 0.01	10/13 (76.9)	0.6	8/60 (13.3)	< 0.01
Tracers						
^99m^Tc	304/390 (77.9)		16/20 (80.0)		10/281 (4.6)	
IDC	69/406 (17.0)	< 0.01	2/2 (100)	0.5	0/66 (0)	0.04
ICG	179/244 (73.4)	0.3	10/11 (90.9)	0.4	4/162 (2.5)	0.9
Single use	4/16 (25.0)		–	–	0/2	–
Double use	165/196 (84.2)	< 0.01	7/9		4/156 (2.6)	
Triple use	173/210 (82.4)	< 0.01	9/11	0.8	6/159 (3.8)	0.9
Surgical method						
Laparoscopy	258/304 (84.9)		6/7 (85.7)		1/243 (0.4)	
Laparotomy	84/118 (71.2)	< 0.01	10/13 (76.9)	0.6	9/74 (12.2)	< 0.01

*BMI* body mass index, ^*99m*^ *Tc* 99m-technetium-labeled tin colloid, *IDC* indigo carmine, *ICG* indocyanine green

## Discussion

In the current study, patients with < 50% myometrial invasion and low-grade tumors not only had higher detection rates, but also had lower FN rates; these patients could avoid systematic lymphadenectomy according to the status of the SLN biopsy. Furthermore, laparoscopic procedures also had a higher detection rate and a lower FN rate than laparotomies. In contrast, we recommended that systematic PLND and PAND should be performed in patients with high-risk endometrial cancers, such as patients with > 50% myometrial invasion or high-grade tumors, as these patients had lower detection rates and higher FN rates.

In the published literature, the detection rate ranged from 62−100% [28, 29], while different injection sites or tracers affected the values. Most investigators described the overall detection rate as at least unilateral detection. The bilateral detection rate ranged from 34−100% [28, 29]. In our study, the detection rate, sensitivity and the FN rate were calculated according to the HP; the overall detection rate and bilateral detection rate were 93.0 and 71.0%, respectively. The injection site was different in each study and included the cervix, myometrial or peri-tumoral hysteroscopy or transvaginal ultrasonography. The overall detection rate after cervical injection ranged from 62−100%, and from 73–95% after corporeal injection [28, 30]. It has been thought that lymph node metastasis initially occurs at the pelvic lymph node basins and secondly in the para-aortic nodes [31], whereas direct lymphatic drainage occurs through the infundibulopelvic ligament or presacral lymphatics directly to the aortic bifurcation [32, 33]; the detection rate of the para-aortic SLN is dependent on the injection site. The para-aortic SLN detection rate was 39% after corporeal injection, 2% after cervical injection, and 17% after deep cervical injection [28]. In our study, the para-aortic SLN detection rate was 6.6%. We do not believe that it is important to identify the para-aortic SLN in patients with low-risk endometrial cancer. In such patients, the SLN could be used for the extent of lymphadenectomy; however, para-aortic lymph node metastasis without pelvic lymph node metastasis is extremely rare. In contrast, the para-aortic SLN as well as the pelvic SLN is important in patients with high-risk endometrial cancer; adjuvant therapy according to ultrastaging of the SLN may result in a favorable prognosis. It is difficult to select the most suitable tracer as it is necessary to take detection ability, ease of use and cost into consideration. However, a combination of either blue dye and Tc or ICG results in the highest detection rate [28, 30]. In the present study, the detection rate was lower in elderly patients. We considered that older patients tended to have type 2 tumors. Elderly patients had a higher rate of type 2 tumors than younger patients (38.3 vs 16.2%). In the current study, the detection rate did not differ between surgeons who had experienced 5 or 10 cases. Based on our experience, we are of the opinion that it takes <5 cases to master this technique.

There are several reports about the relationship between myometrial invasion or tumor grade and the accuracy of the SLN biopsy. Naoura et al. reported the relationship between the European Society of Medical Oncology (ESMO) risk group and accuracy of the SLN [34]. In this study, the FN rate was 6% for the whole population. The rate was significantly higher for patients with unilateral SLN detection and for those in the high-risk group compared with those in the low/intermediate risk group (2.3 vs 20%). When excluding patients with unilateral SLN detection, the FN rate was 3% in the whole population and 9% for patients in the high-risk group. There was no difference in the detection rate according to the presumed type of endometrial cancer, presumed risk group or final histology according to the ESMO risk group [34]. In the Senti-Endo trial, no FN cases were observed in type 1 endometrial cancer; however, 18% of cases were FN in type 2 endometrial cancer [29].

Complete surgical staging, including hysterectomy, bilateral salpingo-oophorectomy, PLND and PAND is recommended for patients with endometrial cancer [1, 35]. However, several studies have shown that the rates of lymph node metastasis and recurrence are extremely low for patients with low-risk endometrial cancer [31, 36–41]. Moreover, recent large studies have revealed no survival advantage of routine lymphadenectomy [42–46], especially in para-aortic lymphadenectomy [14], for patients with low-risk disease; full surgical staging including systematic lymphadenectomy is not recommended in these patients [10]. In contrast, systematic lymphadenectomy including PLND and PAND has resulted in a favorable prognosis for patients with high-risk disease [14]. Our data suggested that systematic lymphadenectomy may not be needed for low-risk patients with no metastasis in the SLN. For high-risk patients, systematic lymphadenectomy including PLND and PAND should be performed regardless of the SLN status. However, SLN mapping itself is important even in high-risk patients because adjuvant therapy according to ultrastaging of the SLN may be performed.

In our study, the detection rate and sensitivity were 84.9 and 85.7%, respectively, for laparoscopic procedures, which is a higher detection rate than for laparotomies. A previous meta-analysis showed that the detection rate and sensitivity were 82 and 96% for laparoscopic surgery, 86 and 90% in robot-assisted surgery, and 77 and 89% for open surgery, respectively [47]. These results demonstrated that laparoscopic and robot-assisted surgery were associated with higher detection rates and sensitivities than open surgery [47]. We believe that the wide and clear view possible with laparoscopy improves the detection rate compared with open surgery; systemic PLND can be omitted in more patients with a higher detection rate under laparoscopy.

This study is associated with three major limitations that may reduce its value. First, the study size was not large enough for multivariate analysis to be performed. Second, the study included bias. For instance, most patients with clinically early disease underwent surgery with laparoscopic procedures. Third, ultrastaging or immunohistochemistry were not performed in all lymph modes. Therefore, our results must be confirmed in further studies.

In conclusion, patients with < 50% myometrial invasion and low-grade tumors not only had higher detection rates, but also had lower FN rates. Although a large randomized clinical trial is required, our data demonstrated that patients with low-risk endometrial cancer who underwent laparoscopic surgery were able to avoid systematic lymphadenectomy according to the status of the SLN biopsy. In contrast, systematic PLND and PAND should be performed in patients with high-risk endometrial cancer, such as high-grade tumors or > 50% myometrial invasion.
